# Retained Catheter Fragment After Continuous Paravertebral Block Placement for Thoracoscopic Repair of Tracheoesophageal Fistula of a Neonate: A Case Report

**DOI:** 10.3390/children13060733

**Published:** 2026-05-25

**Authors:** Roshni Cheema, Mihaela Visoiu

**Affiliations:** Department of Anesthesiology and Perioperative Medicine, University of Pittsburgh Medical Center, Children’s Hospital of Pittsburgh, 4401 Penn Ave., Pittsburgh, PA 15224, USA; cheemar5@upmc.edu

**Keywords:** neonates, paravertebral nerve block, catheter breakage, complication

## Abstract

**Highlights:**

**What are the main findings?**

- Retained paravertebral catheter fragments in neonates are a rare but important complication that can present diagnostic and management challenges.
- Plain radiographs can help to identify and localize retained fragments.

**What are the implications of the main findings?**

- Catheter integrity should always be verified before and after placement, with careful documentation of its handling.
- Small, non-metallic fragments away from critical structures may be managed conservatively, alongside early transparent communication with caregivers.

**Abstract:**

Background: Thoracic paravertebral catheters are increasingly used in neonates to avoid neuraxial techniques during thoracoscopic tracheoesophageal fistula (TEF) repair. Catheter fracture and retention are exceedingly rare in this population, and optimal management remains undefined. Learning Objectives: Recognize this complication risk in neonatal paravertebral placement; identify appropriate imaging when retention is suspected; discuss conservative and surgical approaches; and understand the importance of early transparent communication with caregivers. Case: A 2-day-old term neonate weighing 2.90 kg underwent thoracoscopic repair of type C tracheoesophageal fistula with intraoperative placement of an ultrasound-guided right paravertebral catheter for continuous analgesia. The catheter was placed at the T5 vertebral level using a 20 G, 2-inch Tuohy needle with an in-plane lateral-to-medial approach. Saline hydrodissection was used to confirm entry into the paravertebral space. A 24 G radiopaque Perifix One catheter was initially inserted but proved difficult to advance. During attempted removal, some resistance was encountered, and both the needle and catheter were withdrawn together. Subsequent inspection suggested possible catheter shortening, raising concern for a retained fragment. A second catheter of size 20 G advanced via an 18 G needle was then successfully placed at the same level and was removed without complications on postoperative day 3. Comparison with an intact reference catheter revealed that the first-placed 24 G catheter was approximately 1.5 cm shorter, although the tip appeared intact. The pain physician promptly notified both the clinical teams and the family. One month later, during routine imaging for respiratory distress, a curvilinear opacity was noted at the T9 vertebral level. Dedicated thoracic spine films confirmed a 7 mm retained paravertebral catheter fragment. Multidisciplinary consensus (pain team, anesthesia, NICU, and surgery) determined that the fragment was small, non-metallic, and remote from critical structures. Conservative management with long-term follow-up was chosen. The family was informed early during initial suspicion and again upon confirmation. At 17-month follow-up, the child remained asymptomatic. Discussion: Retained catheter fragments are rare in pediatric regional anesthesia and may be radiographically occult early. In neonates, re-operation for a tiny, inert foreign body may cause more morbidity than observation. Prevention depends on appropriate equipment selection, catheter integrity checks pre- and post-placement, careful technique, and attention to resistance or difficulty during advancement or removal. Clear and timely communication with caregivers preserves trust when complications or iatrogenic uncertainty arise. Conclusions: In this neonate, a small retained paravertebral catheter fragment was identified incidentally and was safely managed with conservative observation. When such fragments are non-metallic, stable, and located away from critical structures, non-operative management with close follow-up may be an appropriate and safe approach.

## 1. Introduction

Paravertebral blocks have become an increasingly important component of postoperative analgesia in pediatric thoracic surgery, as they offer a means to avoid the risks associated with neuraxial techniques [1,2,3,4]. In neonates, regional anesthesia techniques that avoid neuraxial instrumentation are often favored due to concerns regarding epidural analgesia-related complications such as bleeding, inadvertent subarachnoid placement, and neuraxial infection [5,6,7]. In addition, epidural procedures are more challenging to perform in this patient population. For thoracoscopic TEF repair in neonates, paravertebral blocks may serve as an alternative to epidural analgesia because they can provide greater hemodynamic stability, avoid potentially serious neuraxial complications, and have better alignment with minimally invasive approaches [2,8].

Ultrasound guidance enhances safety and efficacy and has been used to place paravertebral catheters during tracheoesophageal fistula (TEF) repair in neonates [1,9,10,11]. Although they occur infrequently, complications such as catheter fracture or retention represent potentially serious events [10,12]. The existing literature on retained paravertebral catheter fragments is sparse and largely limited to isolated case reports in older pediatric patients and adults [12,13,14,15,16]. These reports highlight variability in presentation, challenges in radiographic identification, and a lack of consensus regarding management strategies.

In this report, a thoracic paravertebral catheter was placed under ultrasound guidance to provide postoperative analgesia following the thoracoscopic repair of a tracheoesophageal fistula in a 2-day-old neonate. During placement, the catheter was removed after difficulty advancing it and was found to be fractured; the retained fragment was later identified at the T9 vertebral level. This case highlights the diagnostic challenges, management strategies, and long-term considerations in a neonate with a retained paravertebral catheter fragment. We aim to contribute to the limited literature in this area and to emphasize practical considerations for prevention, detection, and communication with caregivers when such rare complications occur.

## 2. Case Report

The patient is a 2-day-old, term male infant (born at 40 weeks + 6 days via normal vaginal delivery), weighing 2.90 kg, scheduled for right video-assisted thoracoscopic repair of type C tracheoesophageal fistula (TEF) with esophageal atresia, under general anesthesia. Additional medical problems included patent foramen ovale, patent ductus arteriosus, mild tracheomalacia, and a borderline type I laryngeal cleft. To provide optimal postoperative analgesia, an ultrasound-guided right paravertebral block catheter was planned to be placed at the end of the surgical procedure while the patient was under general anesthesia.

**Figure 1:**
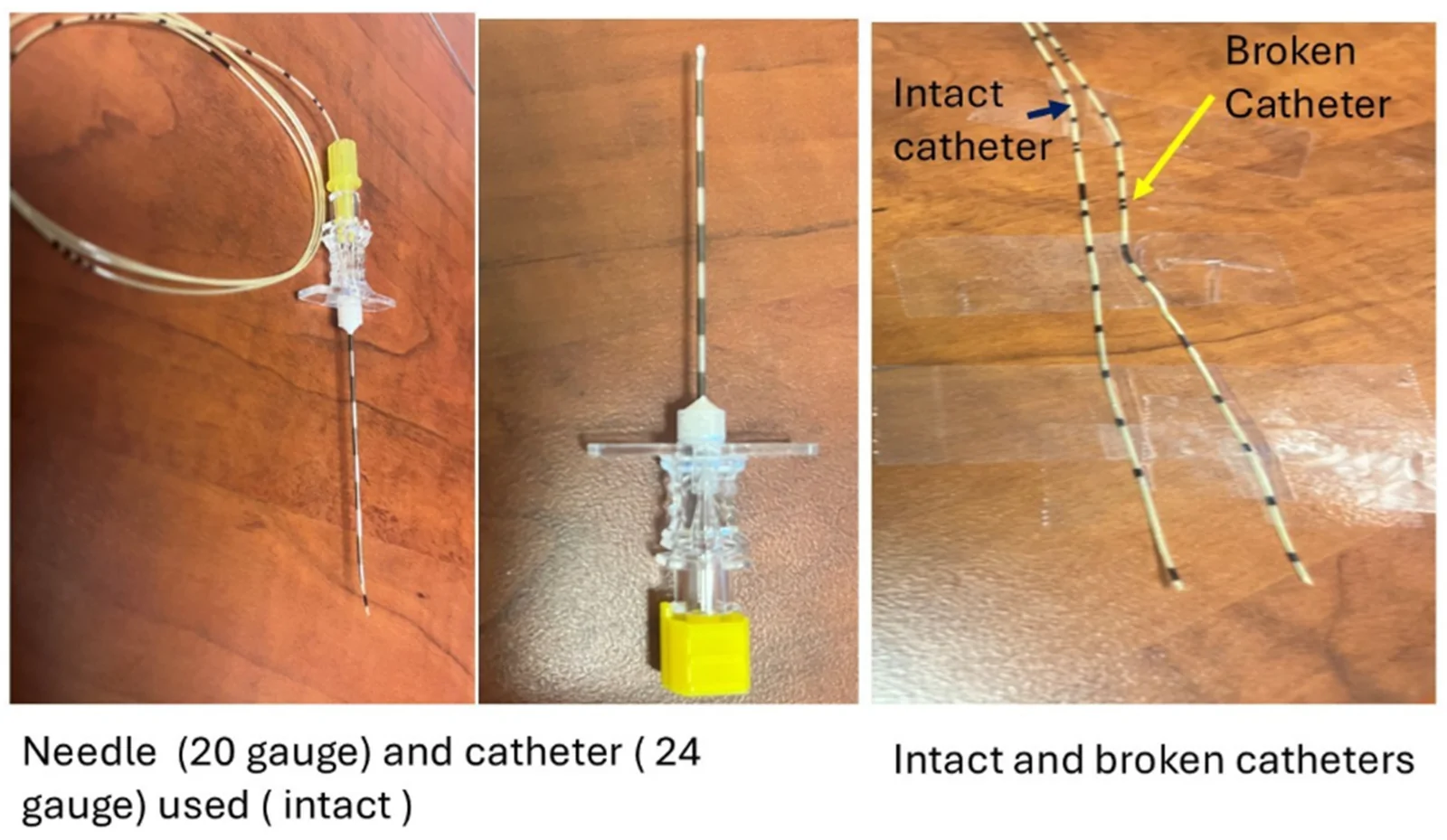
Intact vs. Broken catheter with needle.

The neonate was intubated using a straight, cuffed, size 3 endotracheal tube, and no complications were encountered during the surgical procedure. A right-sided chest tube was placed at the end of the surgery. The neonate was maintained in the right lateral position for placement of a nerve block catheter. Using sterile technique, the T5 transverse process, pleura, and spinal cord were visualized with a high-frequency hockey-stick transducer placed in a transverse-oblique orientation. Under direct visualization, a 5 cm, 20-gauge Tuohy needle (Figure 1) (B. Braun Medical Inc., Bethlehem, PA, USA) was inserted in-plane from the lateral aspect of the transducer toward the visualized transverse process. It was confirmed in the paravertebral space by anterior displacement of the pleura after injection of 1 mL of saline. An additional 1 mL of 0.2% ropivacaine was administered after negative aspiration. A 24-gauge catheter (Figure 1) (24 Ga × 72 cm, closed tip with six lateral ports PERIFIX ONE Epidural Catheter, B. Braun Medical Inc., Bethlehem, PA, USA) was threaded via the needle; however, the catheter could not be advanced much beyond the needle tip. The decision was to remove the catheter; however, due to some resistance, both the needle and the catheter were removed simultaneously. The catheter was inspected, and the tip appeared intact, as evidenced by the present distal marking. Due to difficulty advancing the 24 G catheter, a larger needle and catheter were chosen for a second attempt. Using a similar technique, a different 20-gauge multi-orifice, closed-tip catheter (EC20C 20 G × 100 cm, Perifix Epidural Catheter, Braun Medical Inc., Bethlehem, PA, USA) was then placed at the same paravertebral level via a 5 cm long, 18-gauge Tuohy needle (PAJUNK^®^, Medical Systems, L.P., GA, USA). Additional saline and ropivacaine 0.2% were injected. It was secured at 4 cm at the skin level. No additional complications were encountered during the placement of the second catheter.

At the conclusion of the nerve block technique, the previously used catheter was inspected again. Although the tip markings appeared intact, the catheter was missing some markings. Compared with an unused reference catheter, the previously used catheter appeared to be approximately 1.5 cm shorter (Figure 1). The previously used needle was examined, but no catheter fragment was found within it. This raised the possibility of a retained fragment from the first used catheter. The teams involved in the patient’s care (surgeon and neonatal intensive care) and the mother were informed of the possibility of catheter breakage during placement. A postoperative chest X-ray was done and reviewed by attending pediatric radiologists, but the missing catheter fragment was not identified on the initial study.

For perioperative pain management, 0.2% ropivacaine (3 mL) was injected via the paravertebral catheter after the surgical procedure.

During the postoperative period, the patient received a continuous infusion of 0.1% ropivacaine at 0.7 mL/h via the paravertebral catheter on the day of surgery. This was tapered to 0.5 mL/h on postoperative day 1. In addition, the patient received acetaminophen intravenously at 40 mg/kg/day and morphine at 0.16 mg/kg/day.

N-PASS (Pain, Agitation, and Sedation Scale) scores remained 0/10 on the day of the procedure and the next two postoperative days. On postoperative day 3, the paravertebral catheter was removed by the acute pain team attending as the dressing got contaminated by the fluid leaked from the chest tube. The catheter tip was intact. No complications from regional anesthesia procedures were encountered. The patient was extubated on postoperative day 4.

**Figure 2:**
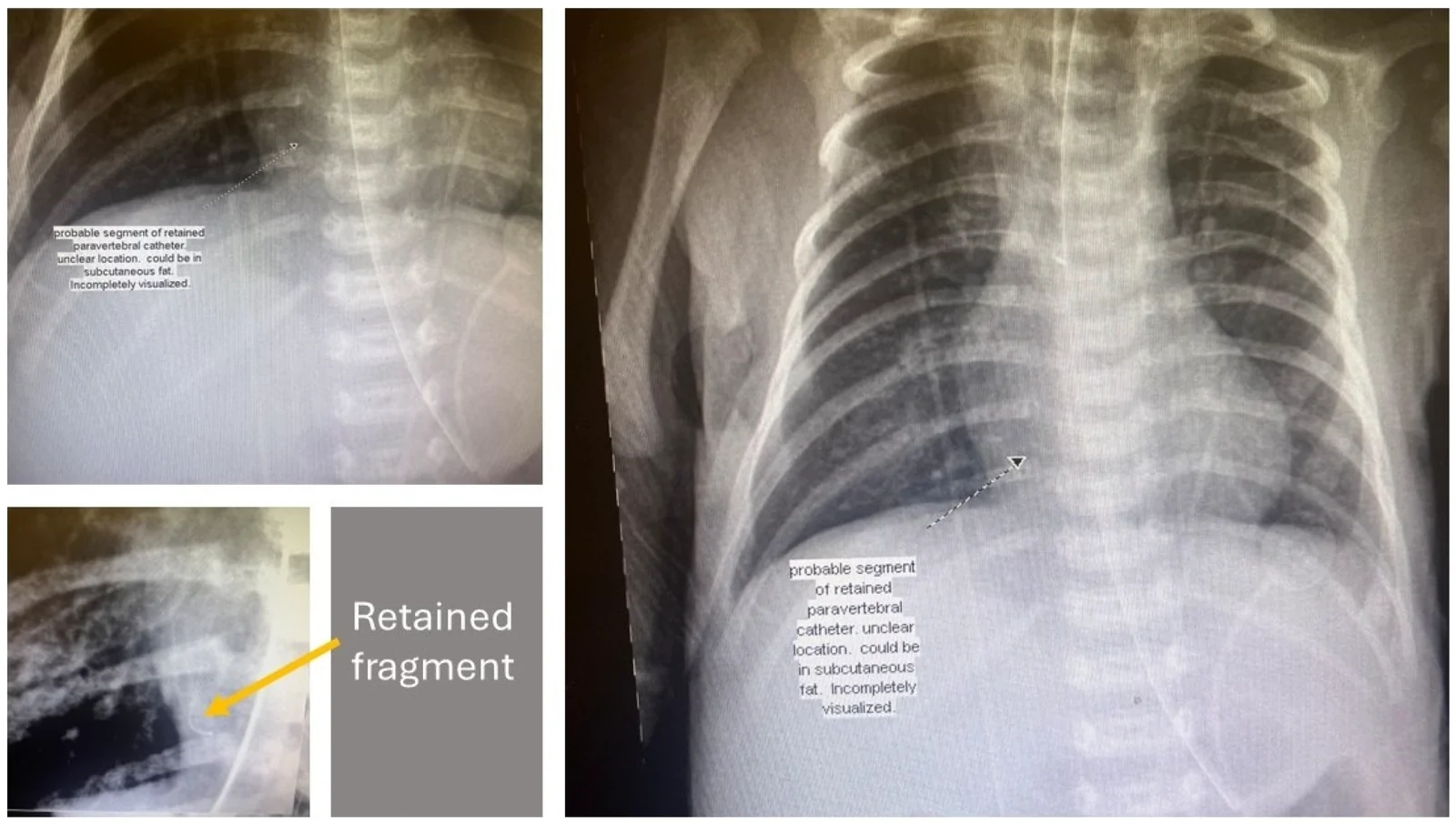
Chest X-ray revealing retained catheter fragment.

One month later, the infant developed an upper rhino/enterovirus respiratory infection associated with feeding-related complications, such as aspiration with oral feeds. Subsequently, he developed respiratory distress that required intubation. During routine chest imaging performed to confirm endotracheal tube position, a curvilinear radiopaque density was noted in the right paravertebral region at the level of T9, raising concern for a retained foreign body (Figure 2). Later, dedicated thoracic spine X-rays (AP and lateral views) confirmed a 7 mm retained paravertebral catheter fragment located anterior and to the right of the T9 vertebral body. There were no signs of abnormal calcifications, infections, or pneumothorax.

The multidisciplinary team, including representatives from pain service, NICU, and pediatric surgery, discussed the findings in depth. The retained fragment was small (7 mm), non-metallic, and located away from critical structures, including the spinal cord, lung parenchyma, trachea, esophagus, and major neurovascular structures. Given the fragment’s stable, asymptomatic nature, the decision was made to manage it conservatively with close follow-up and observation. The mother was promptly informed of the findings, and a thorough discussion regarding the risks, uncertainties, and the long-term plan was held. Symptoms associated with potential complications (back pain, localized infection, hematoma) were mentioned to her. She was notified that the missing catheter fragment was not located inside the spinal canal. She was reassured that removal was not recommended unless complications arose in the future.

On follow-up, there were no neurological deficits or signs of local inflammation. The patient continued to be monitored for TEF-related complications, including feeding difficulties and respiratory status. The retained catheter fragment remained asymptomatic, and no further intervention was required. At the most recent follow-up at 17 months of age, clinical assessment by the pain team confirmed that the child remained asymptomatic with no complications related to the retained catheter fragment. Table 1 shows the timeline of events from the day of surgery till the last follow-up.

## 3. Discussion

Retained foreign bodies, including catheter fragments, are a rare but significant complication in pediatric patients undergoing regional anesthesia. While paravertebral blocks are effective in providing analgesia in pediatric thoracic surgery, their use in neonates requires particular care, given the anatomical challenges and the risk of catheter-related complications [9,12].

This case highlights the rare but important complication of a paravertebral catheter placed in neonates. Continuous paravertebral catheter techniques were selected in this case because thoracoscopic tracheoesophageal fistula repair is associated with significant postoperative pain extending beyond the expected duration of a single-injection block. Continuous catheter-based analgesia allows prolonged opioid-sparing analgesia, titrable local anesthetic administration, and avoidance of repeated systemic opioid dosing in a fragile neonatal population. However, their small anatomical size, the lack of appropriate needle and catheter sizes, and the limited opportunities to place paravertebral catheters make this technique challenging to perform. In addition, limited familiarity with all potential complications makes it more difficult for providers to manage them effectively and to communicate clearly with families and other clinicians about both immediate and long-term outcomes.

Breakage of a paravertebral catheter is a rare event. It has been reported in pediatric and adult cases [13], but to our knowledge, the literature on neonates is very limited [12,14]. It may result from various causes, including technical errors during insertion or withdrawal, inappropriate equipment (e.g., a sharp needle aperture or a small-gauge catheter), or catheter manufacturing defects.

In this case, the exact mechanism is uncertain. An experienced pediatric pain physician performed the procedure. One possible explanation is that during advancement, the catheter became pinched between the tip of the Tuohy needle and the transverse process, potentially at one of the needle apertures, preventing further advancement. Although the needle and catheter were removed simultaneously, the catheter may have been stretched and subsequently sheared by the needle’s cutting edges. It is also important to note that the aperture edges of a 20-gauge needle are sharp, and the opening at the tip of the Tuohy needle is narrower than the needle itself. This configuration can create resistance during catheter manipulation, regardless of the needle’s position relative to adjacent bony structures.

The catheter breakage was not identified immediately. The 24-gauge catheter tip markings are different from those of the 20-gauge catheter, and these smaller devices are used infrequently. These small needles and catheters were selected because they were considered more appropriate for the infant’s weight and age. However, the differences in catheter markings and device characteristics may have contributed to the provider not immediately recognizing that the catheter was missing part of its tip.

The size of the identified retained paravertebral catheter fragment was smaller than the presumed size of the missing fragment based on measurements with an unused catheter. The 24-gauge catheter is easily stretchable, which could occur during removal. In addition, the catheter size and integrity were not verified before placement, and the catheter length may be shorter than expected, as previously reported [12].

Plastic fragments are difficult to visualize on X-ray, as described by a few case reports [12].

Visoiu et al. [12] reported a breakage of a paravertebral catheter during placement (Table 2) in a pediatric patient. The 1.5–1.8 cm missing fragment could not be visualized by ultrasound, fluoroscopy, computed tomography, or a spine magnetic resonance scan or cut-down [12]. Sihoe et al. [14] reported a catheter placed by thoracotomy and broken during removal (Table 2). The piece could not be localized using computed tomography [14].

In our case, the missing fragment could not be visualized on the initial radiographs, but it was identified one month later. It may have been missed on the initial radiograph due to the presence of multiple lines, chest tubes, electrodes, and cables used to monitor the infant. After the fragment was discovered, a second review of the initial radiograph identified the missing fragment in the same location, at the T9 level. It is worth noting that the fragment was not found at the level of catheter insertion, the T5 level. It was observed at a location lower than expected. As mentioned before, the second catheter was placed at the same vertebral level, and it is a possibility that the saline and medication injected via the needle may have contributed to caudal migration of the fragment to a lower paravertebral level. The location of the missing fragment suggests that both catheters were within the paravertebral space.

The optimal management of a broken paravertebral catheter remains unclear. Visoiu et al. [12] tried to remove the small fragment using a cut-down. Their decision was supported by the potential side effects of chemotherapy (immunosuppression and coagulopathy), with an increased risk of abscess formation and bleeding around the missing piece [12]. However, the piece could not be removed.

After the first radiography, we were not sure if the missing fragment was left inside the neonate’s body. However, once we identified it, we did not attempt to remove the fragment. The small size and non-metallic composition of the retained fragment in this case, combined with its location away from the posterior mediastinum and other structures, made conservative management the most appropriate course of action. In addition, the fragment did not move from its initial location, which was reassuring.

However, in some cases, retained catheter fragments can cause complications such as pain, focal tenderness, abscess, or hematoma. Fujii et al. described the case of a 65-year-old woman with lung cancer who had a 100 mm catheter fragment retained (Table 2) for 2 years that was not identified on initial chest imaging. When the patient reported focal tenderness, further evaluation with a computed tomography scan revealed the fragment [15]. The catheter was later surgically removed due to the length of the fragment, resultant coagulopathy, and immunosuppression from chemotherapy, which increased the risk of hematoma and abscess formation [15].

Effective management of such complications requires clear and open communication with caregivers. Retained paravertebral catheter fragments are rare, and reports in neonates are lacking. The full range of potential complications in this population is not well established. In this case, the medical team informed the neonate’s mother early and provided reassurance regarding the low risk of harm. The pain physician openly acknowledged the complication and apologized. The mother expressed understanding, and this transparent communication helped maintain trust throughout the course of management.

## 4. Conclusions

To our knowledge, reports of paravertebral catheter breakage in neonates are extremely limited. Providers performing this procedure should be familiar with the characteristics of needles and catheters available for neonatal use. While initial imaging may miss a retained fragment, repeat imaging, particularly if clinical suspicion persists, can facilitate identification. Conservative management with long-term follow-up may be appropriate in carefully selected cases, whereas surgical removal of the fragment should be considered based on the patient’s condition and associated risks. Given the rarity of this complication and the limited data in neonates, the full spectrum of potential outcomes remains uncertain. Therefore, transparent and early communication with caregivers is essential to preserve trust and to guide management decisions in cases of iatrogenic complications.

## Figures and Tables

**Table 1:** Timeline of events.

Timepoint	Event
Day 0	Initial catheter placement was attempted, followed by removal due to suspicion of catheter shortening; postoperative X-ray was negative. A second catheter was placed shortly after, without complications
Postoperative Day 3	The second catheter was removed and confirmed to be intact
1 Month	A retained catheter fragment was incidentally detected on imaging performed for TEF-related care; dedicated thoracic spine X-rays (AP and lateral views) confirmed its location
3 Months	Follow-up showed the patient remained asymptomatic with no complications related to the retained catheter fragment
6 Months	Follow-up confirmed that the patient remained asymptomatic without complications
12 Months	Ongoing follow-up demonstrated no symptoms or complications related to the retained catheter fragment
17 Months	The latest follow-up showed the patient remained asymptomatic

**Table 2:** Selected published case reports of Broken/Retained Paravertebral catheters.

Author/Year	Patient Age/Gender	Surgery	Location of PVC	Broken During Placement vs. Removal	Length of Fragment	Radiographic Evidence	Attempt to Remove
Sihoe et al. (2008) [14]	34/M	Rt upper lobectomy	Sub-pleural pocket (3rd–6th ICS)	Removal	13 cm	No	Yes, removed by VATS as long fragment left behind
Fujii et al. (2017) [15]	65/F	Lower lobectomy	5th thoracic vertebral body	Placement	10 cm	Yes, CT scan	Yes, removed as patient symptomatic
Saeki et al. (2017) [13]	50/M	Lower lobectomy	-	Removal, but broke intraoperatively due to electrocautery	4 cm	Not done	Yes, removed in same surgical setting
Agrafiotis et al. (2018) [16]	62/F	Rt lower lobectomy	Extrapleural space (3rd ICS)	Removal	1 cm	Yes, Chest X-ray and CT scan	No
Visoiu et al. (2021) [12]	3/M	Exploratory laparotomy	T5	Placement	1.9 cm	-	Yes, but could not be retrieved

## Data Availability

The original contributions presented in this study are included in the article. Further inquiries can be directed to the corresponding author.
